# Heparanase Overexpression Reduces Hepcidin Expression, Affects Iron Homeostasis and Alters the Response to Inflammation

**DOI:** 10.1371/journal.pone.0164183

**Published:** 2016-10-06

**Authors:** Michela Asperti, Tanja Stuemler, Maura Poli, Magdalena Gryzik, Lena Lifshitz, Esther G. Meyron-Holtz, Israel Vlodavsky, Paolo Arosio

**Affiliations:** 1 Molecular Biology laboratory, Department of Molecular and Translational Medicine DMMT, University of Brescia, Viale Europa 11, 25123, Brescia, Italy; 2 Laboratory for Molecular Nutrition, Faculty of Biotechnology and Food Engineering, Technion-Israel Institute of Technology, Technion City, 32000, Haifa, Israel; 3 Cancer and Vascular Biology Research Center, Rappaport Faculty of Medicine, Technion- Israel Institute of Technology, Haifa, 31096, Israel; CINVESTAV-IPN, MEXICO

## Abstract

Hepcidin is the key regulator of systemic iron availability that acts by controlling the degradation of the iron exporter ferroportin. It is expressed mainly in the liver and regulated by iron, inflammation, erythropoiesis and hypoxia. The various agents that control its expression act mainly via the BMP6/SMAD signaling pathway. Among them are exogenous heparins, which are strong hepcidin repressors with a mechanism of action not fully understood but that may involve the competition with the structurally similar endogenous Heparan Sulfates (HS). To verify this hypothesis, we analyzed how the overexpression of heparanase, the HS degrading enzyme, modified hepcidin expression and iron homeostasis in hepatic cell lines and in transgenic mice. The results showed that transient and stable overexpression of heparanase in HepG2 cells caused a reduction of hepcidin expression and of SMAD5 phosphorylation. Interestingly, the clones showed also altered level of TfR1 and ferritin, indices of a modified iron homeostasis. The heparanase transgenic mice showed a low level of liver hepcidin, an increase of serum and liver iron with a decrease in spleen iron content. The hepcidin expression remained surprisingly low even after treatment with the inflammatory LPS. The finding that modification of HS structure mediated by heparanase overexpression affects hepcidin expression and iron homeostasis supports the hypothesis that HS participate in the mechanisms controlling hepcidin expression.

## Introduction

Iron is an essential micronutrient for life and its inappropriate levels can lead to harmful effects on the organism [1]. Thus, iron homeostasis needs to be tightly regulated. Hepcidin, a peptide hormone highly expressed in the liver, acts by degrading ferroportin, the major cellular iron exporter. Reduced ferroportin levels limit iron uptake and recycling, consequently hampering systemic iron availability [2]. The regulation of hepcidin expression occurs mainly at transcriptional levels and depends on several factors including iron abundance and utilization, inflammation, hypoxia, and erythropoietic activity. Hepcidin transcription is induced mainly by the Bone Morphogenetic Proteins (BMPs)/SMAD signalling pathway, which involves SMAD1/5/8 phosphorylation, association with SMAD4 and transfer of the complex to the nucleus. The iron-regulated BMP6 interacts with BMP-receptors and co-receptors of hepatic cells to activate the signalling and the BMP-RE element in the hepcidin promoter [3]. The membrane complex that responds to the signalling is known to involve various proteins, including BMP6, BMP receptors, the co-receptor hemojuvelin (HJV), the protease TMPRSS6, neogenin, HFE and TFR2 [4, 5]. Genetic alterations of this pathway can lead to either iron deficiency (e.g. IRIDA) or iron overload (e.g. hemochromatosis). Hepcidin is also upregulated by the inflammatory cytokine IL-6, via STAT3 signaling [6], thus contributing to the pathogenesis of the anemia of chronic diseases (ACD) [7]. We previously demonstrated that exogenous heparins interfere with the BMP/SMAD pathway and inhibit hepcidin expression *in vitro* and *in vivo* [8], an activity retained by heparin analogues that have been modified to abolish anti-coagulant activity [9].

Heparins have a structure similar to that of heparan sulfate (HS) saccharide chains that are part of the ubiquitous heparan sulfate proteoglycans (HSPGs). HSPGs are involved in several physiological and pathological processes by acting as receptors or co-receptors for circulating proteins [10]. HSPGs are involved also in the BMP-pathway, which is activated by the formation of a multi-molecular complex that may be facilitated by membrane resident molecules, such as the HSPGs. Indeed, HSPGs have been shown to modulate the osteogenic activity of BMP2 and BMP4 [11] by acting as BMP co-receptors [12]. The possible role of the endogenous HS on hepcidin expression and iron homeostasis has not been explored so far. To this aim, we initially searched for enzymes that regulate HS biosynthesis or degradation and among them, heparanase 1 (HPA) plays a major role in HS degradation and in altering HS structure. HPA is an endo-β-D-glucuronidase that is responsible for the cleavage of HS side chains at a limited number of sites, remodeling cell surface and extracellular matrix (ECM) [13, 14]. This HS-degrading enzyme is synthesized as a latent 65 kDa precursor, which first interacts with cell surface HSPGs, then is rapidly internalized and undergoes proteolytic cleavage and maturation in the lysosomes by cathepsin L, generating the active enzyme composed of a 50 kDa and an 8 kDa subunit [15–17]. HPA1 has higher expression during embryogenesis, as well as in cells of the developing vascular and nervous system. Several studies have demonstrated that HPA1 is up-regulated in most human tumors, where it correlates with the metastatic potential and neovascularization of the tumor, and also in inflammation, wound healing, and diabetic nephropathy [18, 19]. Transgenic mice overexpressing human HPA1 (TG-HPA) have been generated and are characterized by lower amounts of HS with altered structure, being short and highly sulfated [20]. These mice are fertile with a normal life span and do not show an overt phenotype. However, they do show an accelerated wound angiogenesis and vascularization, mild kidney damage, increased embryo implantation, and enhanced rate of hair re-growth [20] [21]. Here we show that they also have impaired iron homeostasis. We report the effects on iron homeostasis of HPA1 overexpression in hepatoma cells and in transgenic mice. They consisted in a reduction of hepatic hepcidin expression even after LPS treatment, with hepatic iron accumulation and alteration of the major indices of iron homeostasis. We conclude that endogenous heparan sulfates have a role in the regulation of hepcidin expression and iron homeostasis.

## Methods

### Hepatoma cells transfection

The human hepatoma cell lines, HepG2, Hep3b and HuH7 (IZSLER, Brescia, Italy) were cultured in minimum essential medium (MEM, Gibco, Life technologies), 10% endotoxin-free fetal bovine serum (FBS) (Sigma-Aldrich), 0.04 mg/mL gentamicin (Gibco), 2 mM L-glutamine (Gibco), and 1 mM sodium pyruvate (Carlo Erba) and maintained at 37°C in 5% CO2. The cells were transfected with empty pcDNA3.1 or pcDNA3.1-HPA vector which encodes the full-length human-HPA1 and is described in [22]. Transfections were carried out with Lipofectamin3000 Reagent (Invitrogen), according to the manufacturer’s instructions. For transient transfection, after 48 h the cells were harvested and the overexpression was evaluated by qPCR and Western blotting. For stable transfection we used only HepG2 cells. 48 h after transfection the complete culture medium was supplemented with 600 μg/ml G418 (Geneticin, Sigma-Aldrich) for selection. After 2 weeks the G418-resistant clones were picked, cultured and analyzed for expression of HPA by qRT-PCR and Western blotting. We selected two clones with different expression of HPA (referred as HPA3 and HPA6) and a control one transfected with empty pcDNA3.1 (referred as MOCK). The stable clones were maintained in 300 μg/ml G418.

### Treatments with heparins, BMP6 and IL6

The HPA3 and HPA6 clones and the control MOCK clone were seeded onto 12-well plates (3x10^5^ cells/well) in MEM with 10% FBS. After 24 h the medium was replaced with 1% FBS together with the glycol-split heparin RO-82, 0.12 μg/mL in the presence or the absence of 10 ng/mL of BMP6 (R&D Systems) and the cells incubated for 6 h, as described in [9]. In other experiments the cells were incubated with IL6 50 ng/mL for 3 h (Relia Tech GmbH) or with 6–12 ng/mL of BMP6 for 6 h (R&D Systems). Then cells were collected for RNA extraction and evaluated for hepcidin expression by qRT-PCR or for protein extraction and evaluated for pSMAD5, pSTAT3 and actin by Western Blotting.

### Animals

All animal experiments were conducted in compliance with the Israeli legislation for animal welfare and approved by the animal committee of the Technion. C57BL/6 wild-type mice (WT, 8 mice) and HPA transgenic (TG-HPA, 12 mice) mice in C57BL/6 background [20] were used, all 14-week-old. To mimic an inflammatory stimulus, six mice for experimental group were treated intraperitoneally (IP) with 1mg/Kg LPS (Lipopolysaccharide, Sigma) or saline as control and euthanized after 6 h. Blood, liver and spleen were kept for analysis.

### RNA preparation and quantitative qRT-PCR

Total RNA was isolated from liver tissues or cells using TRI Reagent (Sigma-Aldrich), according to the manufacturer’s instruction. cDNA was generated by Reverse transcription, using 1 μg RNA and qScript cDNA Synthesis kit (Quanta, Bioscience Inc) in 20 μL. Samples were analyzed by quantitative reverse transcription polymerase chain reaction (qRT-PCR), using Perfecta mix reaction ROX Form Quanta (Bioscience Inc.), according to the manufacturer’s instructions. All data are normalized to Hprt1 expression and expressed as Relative Quantification (method of 2^−ΔΔCt) or as −ΔCt (i.e., Ct Hprt − Ct target, the higher is the −ΔCt, the greater is the amount of target amplicon). Primers used for human cells were: Hs Hamp1 forward, 5’-CCA-GCT-GGA- TGC-CCA-TGT-T-3’, and reverse, 5’-GCC-GCA-GCA-GAA-AATGCA- 3’; Hs Hprt1 forward, 5’-TGC-TTT-CCT-TGG-TCA-GGC-AG-3’, and reverse, 5’-AAG-CTT-GCG-ACC-TTG-ACC-AT-3’; Hs Id1 forward, 5’-GTA-AAC-GTG-CTG-CTC-TAC-GAC-ATG-A-3’, and reverse, 5’-AGCTCC-AAC-TGA-AGG-TCC-CTG-A-3’; Hs Soc3 forward 5’-CAG-GAA-TGT-AGC-AGC-GAT-GGA-A-3′ and reverse 5′-CCT-GTC-CAG-CCC-AAT-ACC-TGA-3′; Hs HPA1 forward, 5’-TAC-CTT-CAT-TGC-ACA-AAC-ACT-G-3’ and reverse, 5’-ACT-TGG-TGA-CAT-TAT-GGA-GGT-T-3’; Hs Fpn forward 5’ ATC-CAT-GTG-CGT-GGA-GTA-CG-3’ and reverse 5’- AGG-GGT-TTT-GGC-TCA-GTA-TCT-TT-3’; Hs TfR1 forward 5’-TTT-CCA-CCA-TCT-CGG-TCA-TC-3’and reverse 5’-GGG-ACA-GTC-TCC-TTC-CAT-ATT-C-3’; Hs Zip14 forward 5’-CCT-GCT-TGG-CTT-ATG-GAG-AA-3’and reverse 5’-CCT-CGC-CAT-ACC-GAT-GTA-TTA-G-3’; Hs Bmp6 forward 5’-GGT-CTC-CAG-TGC-TTC-AGA-TTA-C-3’ and reverse 5’-CAG-GTC-TTG-GAA-ACT-CAC-ATA-CA-3’.

The primers for qPCR assay for mouse liver tissues were: Mm Hamp1 forward, 5’-AAG-CAG-GGC-AGA-CAT-TGC-GAT-3’, and reverse, 5’-CAG-GAT-GTG-GCT-CTA-GGC-TAT-GT-3’; Mm Hprt1 forward, 5’-CTG-GTT-AAG-CAG-TAC-AGC-CCC-AA-3’, and reverse, 5’-CAGGAG-GTC-CTT-TTC-ACC-AGC-3’; Mm Id1 forward, 5’-ACC-TG-AACGGC-GAG-ATC-A-3’, and reverse, 5’-TCG-TCG-GCT-GGA-ACA-CAT-G-3’; Mm Bmp6 forward, 5’-GAA-CCT-GGT-GGA-GTA-CGA-CAA-3’, and reverse, 5’-TCC-TTG-TAG-ACG-CGG-AAC-TC-3’; Mm TfR1 forward, 5’- AGC-CAG-ATC-AGC-ATT-CTC-TAA-C3’, and reverse, 5’-TCT-GCA-GCC-AGT-TTC-ATC-TC-3’; Mm Fpn forward, 5’- CAT-TGG-TGA-CTG-GGT-GGA-TAA-G-3’, and reverse, 5’-CAG-GAG-CTC-ATT-CTT-GTG-TAG-G-3’; Mm Zip14 forward, 5’- CAT-GGA-CCG-CTA-TGG-AAA-GAA-3’, and reverse, 5’- CCT-TGG-GCT-GGG-AAA-CAT-TA-3’; Mm Socs3 forward, 5’-TTA-AAT-GCC-CTC-TGT-CCC-AGG-3’, and reverse, 5’ TGT-TTG-GCT-CCT-TGT-GTG-CC-3’; Mm Crp forward, 5’-GCT-ACT-CTG-GTG-CCT-TCT-GAT-CA-3’, and reverse, 5’-GGC-TTC-TTT-GAC-TCT-GCT-TCC-A-3’.

### Tissues or cell lysates and Immunoblot analysis

Liver, spleen or cells extracts were prepared using RIPA buffer (150 mM NaCl, 1% NP-40, 0.5% sodium deoxycholate, 0.1% SDS, 50 mM Tris pH8, 50 mM DTT, 0.01 mg/mL Leupeptine, Protease inhibitor cocktail-Roche) or lysis buffer (200 mM Tris-HCl pH 8, 100 mM NaCl, 1 mM EDTA, 0.5% NP-40, 10% glycerol, 1 mM sodium fluoride, 1 mM sodium orthovanadate; Complete Protease Inhibitor Cocktail; Sigma) and proteins were quantified by Bradford assay (Biorad). Samples of 40–50 μg proteins were separated by 10–14% SDS-PAGE or 8% non-denaturing PAGE and transferred to Cellulose Nitrate Membrane (Whatman) or Hybond-P Membrane (GE). The primary antibodies used for immunoblotting were anti-L-ferritin (SIGMA #F5012), anti-GAPDH (ORIGENE #TA802519), anti-phospho-SMAD5 (ABCAM RabMab #AB92698), anti-SMAD5 (Cell Signalling #9517), anti-phospho-STAT3 (Cell Signaling #9138S), anti-stat3 (Cell Signalling #39132), anti-Actin (ORIGENE #TA890010), anti-TfR1 (Invitrogen #136800), anti-Ferroportin (Abnova, Novus Biologicals #NBP1-21502); anti mouse L-ferritin and anti-HPA #1453, [15]. After incubation with horseradish peroxidase-conjugated secondary antibodies, membranes were developed with SuperSignal West Pico Chemiluminescent Substrate (Thermo scientific-Pierce) and visualized with ImageQuant LAS 4000 device (GE) or Lycor (Odyssey). Band intensity was quantified by densitometry analysis using ImageJ software.

### Iron quantification

Liver and spleen iron content was determined spectrophotometrically as in [23] with minor modifications. Briefly, 50 mg tissues were incubated 18 h in acid solution (3 M HCl, 0.6 M trichloroacetic acid) at 65°C. Samples of 10 μL clarified acid extract were added to 240 μL of working chromogen reagent in a 96 well/plate (1 volume of 0.1% bathophenanthroline sulfate and 1% thioglycolic acid solution, 5 volumes of water, and 5 volumes of saturated sodium acetate). The solutions were then incubated for 30 minutes at room temperature and the absorbance measured at 535 nm in plate reader. A standard curve was prepared with a pre calibrated water solution of FeCl_3_ (Sigma). Blood was collected from the tail and serum iron determined with a commercial kit (Serum Iron Kit, Randox Laboratories, LtD), according to the manufacture’s instruction.

### Serum hepcidin protein content

Mouse serum hepcidin1 (Hepc1) was quantified using Surface Enhanced Laser Desorption Ionization Time of Flight- Mass Spectrometry (SELDI-TOF) as previously described [24, 25].

### Prussian blue staining

Liver and spleen homogenates were pretreated at 70°C for 10 min to enrich the thermostable ferritins. Than samples equivalent to 50 μg of pretreated proteins were loaded on 8% non-denaturing gel and run for 3 h at 160 V. Then, the gels were washed with water and incubated in 2% ferrocyanide, 2% HCl for 1 h at RT. To enhance the signal, the gels were incubated in 0.025% DAB (3,3'-Diaminobenzidine-Sigma) and 0.05% H_2_O_2_ in 20 mM Tris HCl, pH 7.4 for 15–30 min. The reaction was blocked with tap water.

### Perl’s stain of spleen sections

Tissues were fixed in 4% paraformaldehyde (Electron microscopy science), dehydrated in ethanol, then washed with xylene and finally submerged in paraffin. Sections were prepared using a microtome (Shandon finesse ME+, Thermo Scientific) and placed on a 37°C heating plate 18 h for dehydration. Paraffin sections were deparaffinised in xylene, rehydrated with ethanol and rinsed in running water and PBS. The slides were then submerged in 3.5% ferrocyanide, 7% HCl for 20 min, rinsed with water and counterstained with Harry's hematoxylin solution. Next, the slides were dehydrated by ethanol and xylene, coverslipped using mounting glue (Eukitt quick-hardening mounting medium, Sigma-Aldrich) and left to dry for 24 h. Image visualization was performed on a Nikon Eclipse 50i microscope 14 using an X-cite series 120 microscope light source system with the software NIS-Elements Microscope Imaging Software.

### Statistics

Data are shown as mean ± standard error of mean (± S.E.), expressed as fold change and represented with Dot-Plot histograms or histograms (GraphPad Prism6, GraphPad Software). Comparison of values between MOCK and cells transiently overexpressing HPA as well as comparison between wild-type and HPA-TG mice were performed by 2-tailed Student t test for unpaired data, whereas comparison between MOCK and stable HPA clones (HPA3 and HPA6 treated or not treated) were performed by one or two-way ANOVA. Multiple comparisons were corrected by Tukey’s test. Differences were defined as significant for P values < 0.05 and P values were showed.

## Results

### Transient overexpression of heparanase in Hepatoma cell lines

**Fig 1:**
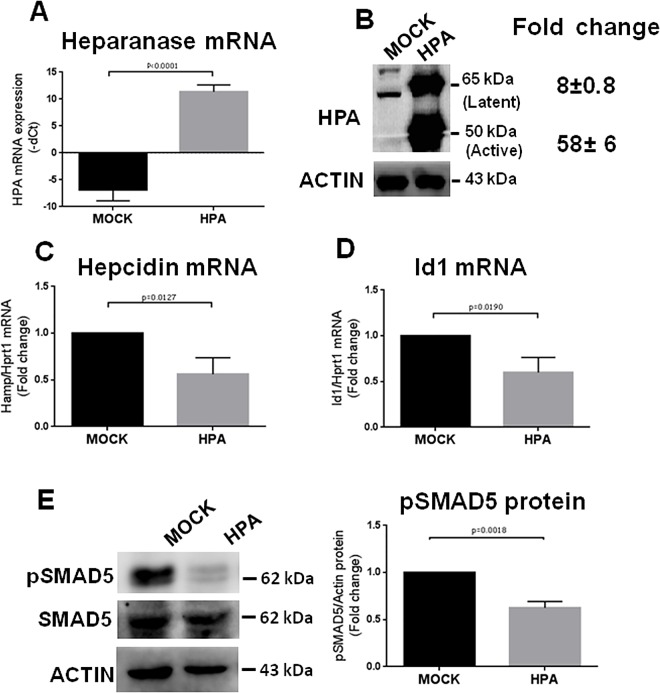
HepG2 cells transiently transfected with heparanase showed a reduction of hepcidin mRNA. HepG2 cells were transfected with pcDNA3.1-HPA plasmid (HPA) or empty pcDNA3.1 as control (MOCK) and harvested 48 h after the transfection. (A) Relative level of HPA mRNA was measured by qRT-PCR (B) Western blot of SDS-PAGE with anti-HPA antibodies show the levels of its latent (65 kDa) and active (50 kDa) form. Densitometry quantification of the two protein forms was performed in relation to Actin. (C) The level of hepcidin mRNA and (D) Id1 mRNA was analyzed by qPCR and normalized for Hprt1. (E) The phosphorylated (pSMAD5) and total SMAD5 were analyzed by western blot and pSMAD5 densitometry was normalized to actin. In (A) the values are expressed as–dCt for HPA mRNA, in C and D as fold change over the control (MOCK) for hepcidin and Id1 mRNA., respectively

The full length human HPA cDNA (HPA) cloned into the pcDNA3.1 vector [22] was transfected in HepG2, HuH7 and Hep3b cells using Lipofectamine3000. Empty pcDNA3.1 plasmid was used as control (MOCK). The cells were harvested 48 h after transfection and RNA and proteins were analyzed. Immunofluorescence staining with anti-HPA antibody showed a transfection efficiency of 60–70% for HepG2 cells, 50% for HuH7 cells and 30% for Hep3b cells (not shown). The initial analyses showed that heparanase was efficiently expressed in the three cell lines and that this was accompanied by a suppression of hepcidin mRNA (Fig 1 and S1A–S1D Fig). Thus, we performed other work only on HepG2 cells, because of our previous experience on this cell type and of its higher transfection efficiency. As expected HPA mRNA was more than 10,000-fold higher in the transfected than in the control cells (Fig 1A) and Western blotting showed that also HPA protein is highly expressed in the transfected cells with a 60-fold increase of the active 50 kDa form and a 8-fold of the latent 65 kDa form (Fig 1B). This was accompanied by a 50% decrease in hepcidin mRNA (Fig 1C). In addition, Id1 mRNA (Fig 1D) and phosphorylated SMAD5 (Fig 1E) two major indices of BMP/SMAD pathway activation were decreased as well. These data indicate that the transient and strong overexpression of HPA affects the BMP/SMAD signaling pathway and hepcidin expression in hepatic cells.

### Constitutive overexpression of heparanase in HepG2 cells

**Fig 2:**
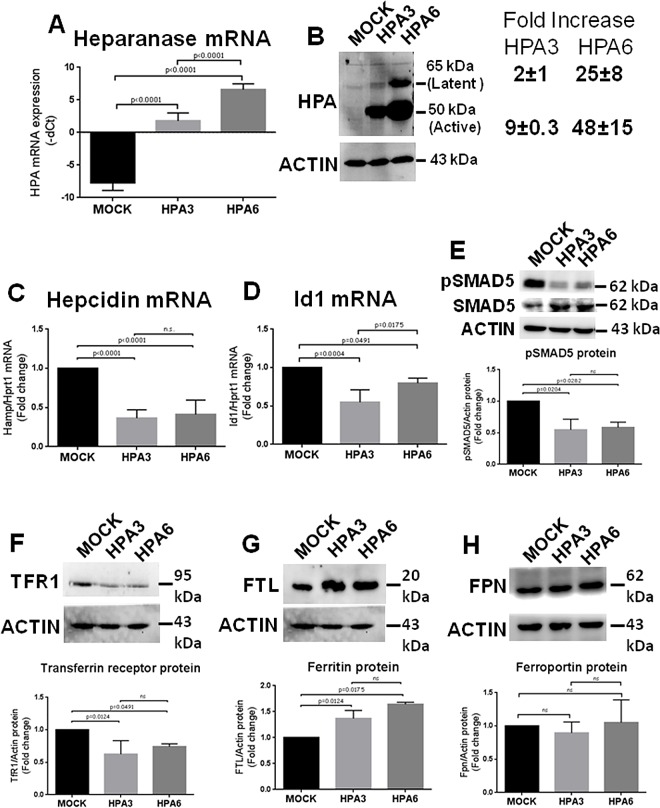
HepG2 clones overexpressing heparanase showed a reduction of hepcidin expression and indices of iron loading. Two stable clones of HepG2 cells transfected with pcDNA3.1-HPA (HPA3 and HPA6) were analyzed for hepcidin expression, BMP/SMAD signaling and indices of iron status. (A) qPCR was assessed to analyze the level of HPA mRNA. (B) Western blot for HPA shows the level of the latent (65 kDa) and active (50 kDa) forms. Densitometry quantification of the two forms was performed in relation to actin as calibrator. (C) qPCR was performed to analyze the level of hepcidin mRNA, (D) and the level of Id1 mRNA in relation to Hprt1. (E) WB of phosphorylated SMAD5 and their densitometry quantification referred to actin and WB of total SMAD5, (F) WB of transferrin Receptor 1, Tfr1, and its densitometry, (G) WB of ferritin light chain, FTL and its densitometry; (H) WB of Ferroportin (FPN) and its densitometry. The values are expressed as–dCt (for HPA mRNA) or as fold change over the control (MOCK) (for hepcidin and Id1 mRNA). The images are representative from three different analyses

To produce stable HepG2 clones, after transfection with pcDNA3.1-HPA construct the cells were selected in G418 (600 *μ*g/ml) for 14 days. We obtained 22 clones, 10 of which were analyzed for HPA expression. We chose one clone with medium and one with high heparanase expression, plus a control one transfected with the empty pcDNA3.1 (MOCK). The high expression clone, coded HPA6, had a major increase of HPA mRNA (Fig 2A) and the 50 kDa active and 65 kDa latent forms of the enzyme increased ~50- and ~25-fold, respectively (Fig 2B). The medium-expression clone, coded HPA3, had a lower increase of HPA mRNA (Fig 2A) and the 50 kDa active and 65 kDa latent forms of the enzyme increased ~10 and ~2-fold respectively (Fig 2B). The two clones showed a similar phenotype with a significant reduction of hepcidin mRNA (50%) (Fig 2C), and Id1 mRNA (20%) (Fig 2D) and inhibition of SMAD5 phosphorylation (Fig 2E). We analyzed also the mRNA level of some iron-related proteins. That of TfR1 decreased of 40–50% in both clones (S2A Fig), while that of Ferroportin increased significantly (S2B Fig). Also the mRNA level of the iron transporter ZIP14 [26] increased in the transfected cells, although it was statistically significant only for the clone HPA6 (S2C Fig). BMP6 mRNA levels didn’t change (S2D Fig). Western blotting of the protein extracts showed that TfR1 level was decreased (Fig 2F), L-ferritin was slightly increased (Fig 2G) while ferroportin protein level was unchanged (Fig 2H).

### Treatment with heparin, BMP6 and IL6

**Fig 3:**
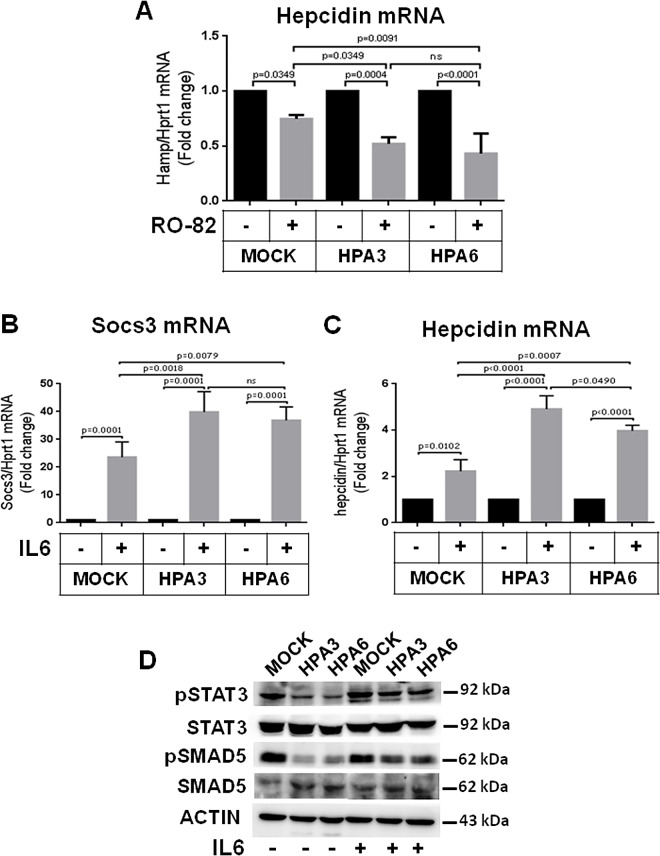
Treatment with heparin and IL6 of HepG2 clones overexpressing heparanase. (A) The two HepG2 clones overexpressing HPA (HPA3 and HPA6) and control (MOCK) cells were treated with 0.12 μg/mL of RO-82 heparin for 6 h and hepcidin mRNA evaluated. (B) The clones and control cells were treated with 50 ng/mL IL6 for 3 h and analyzed for mRNA level of Socs3 and (C) hepcidin in relation to Hprt1. The values are expressed as fold change of their respective untreated controls (-). (D) Western blot of pSMAD5, total SMAD5, pSTAT3, total STAT3 and of ACTIN as housekeeping.

Overexpression of HPA renders the endogenous HS shorter and more sulfated [14]. To verify whether these modifications affected the hepcidin response to heparins the two clones and control cells were treated with a low dose of RO-82 heparin (0.12 μg/mL), in the absence or presence of BMP6 stimulation (10 ng/mL) for 6 h. This concentration of heparin had a minor effect on mock cells, as expected, and stronger inhibitory effect on hepcidin expression in the two clones (Fig 3A and S3A Fig), a difference that vanished in the presence of BMP6 (S3A Fig). A 6 h treatment with 6–12 ng/mL of BMP6 caused a similar induction of hepcidin expression in the MOCK and clones (S3B Fig). The pro-inflammatory cytokine IL6 is known to induce hepcidin. To verify if HPA expression modifies its response, we treated the clones with 50 ng/mL of IL6, for 3 h. Upregulation of Soc3 mRNA confirmed that the treatment was effective, and the response to IL6 was higher in the HPA clones (Fig 3B). Hepcidin mRNA response to IL6 was about double in the HPA3 and HPA6 clones compared to control (Fig 3C). Western blot showed that pSTAT3 and pSMAD5 increased in the two clones after the IL6 stimulation (Fig 3D). These data indicated that HPA overexpressing cells were more sensitive to inflammatory stimuli, to which they responded with higher hepcidin expression.

### Hepcidin expression in heparanase transgenic mice

**Fig 4:**
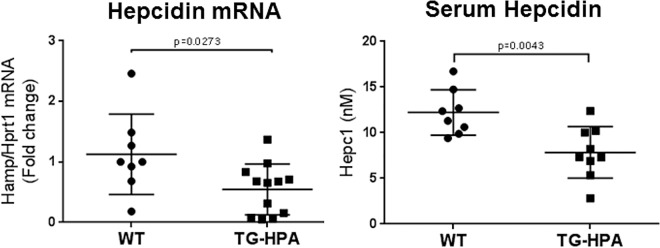
Transgenic mice overexpressing heparanase showed reduced liver hepcidin mRNA and serum protein. (A) Liver hepcidin mRNA levels of wild type (WT) and transgenic HPA mice (TG-HPA) normalized to Hprt1. The values are expressed as fold change of wild type mice. (B) Quantification of serum hepcidin by SELDI-TOF in the same mice.

The data of transfected cells showed that up-regulation of HPA was accompanied by a repression of hepcidin level and a higher response to inflammatory stimuli. Next we investigated if this occurred also in mice overexpressing human HPA. We analyzed 14-week old male TG-HPA [20] and matched control mice (10–12 mice/group). Liver hepcidin mRNA level was about 50% lower in TG-HPA mice compared to controls (Fig 4A), and hepcidin protein in serum was similarly reduced (Fig 4B).

### Transgenic mice over-expressing heparanase have an altered iron homeostasis

**Fig 5:**
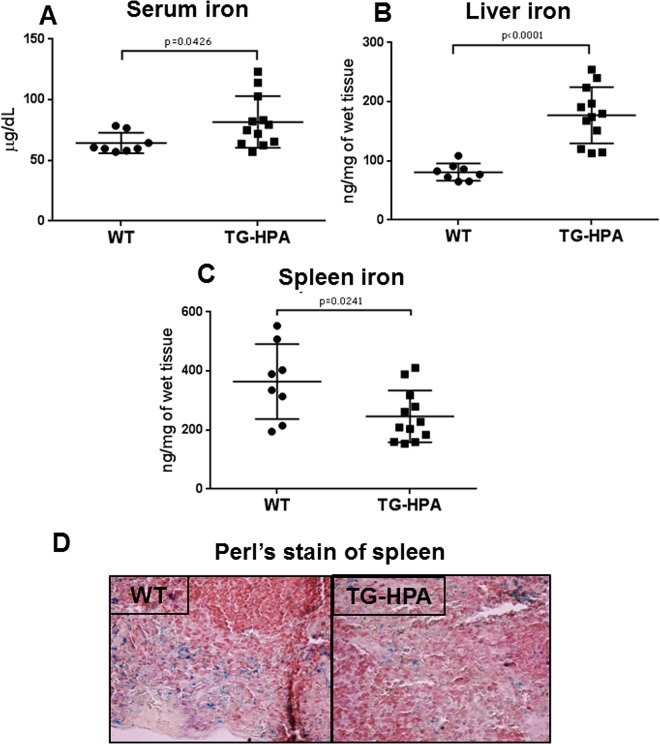
Transgenic mice overexpressing heparanase showed altered iron homeostasis. (A) Levels of serum iron in the WT and TG-HPA mice, (B) non-heme liver iron and (C) non-heme spleen iron levels, measured by a spectrophotometric assay. (D) Perl’s stain of spleen sections that showed a lower number of iron granules in TG-HPA compared to WT mice. The sections were counterstained with hematoxylin and eosin

Low levels of liver hepcidin expression are expected to deregulate systemic iron homeostasis. In fact we found that in the TG-HPA mice serum iron was significantly higher compared to controls (80 μg/dL TG-HPA *vs* 60 μg/dL WT) (Fig 5A) and the same tendency was detected for liver iron (180 ng/mg TG-HPA *vs* 80 ng/mg WT) (Fig 5B). In contrast spleen iron was significantly lower (280 ng/mg TG-HPA *vs* 360 ng/mg WT) (Fig 5C) in TG-HPA mice, a finding supported by the lower number of Perl’s-positive granules in spleen slices of the TG-HPA mice (Fig 5D).

**Fig 6:**
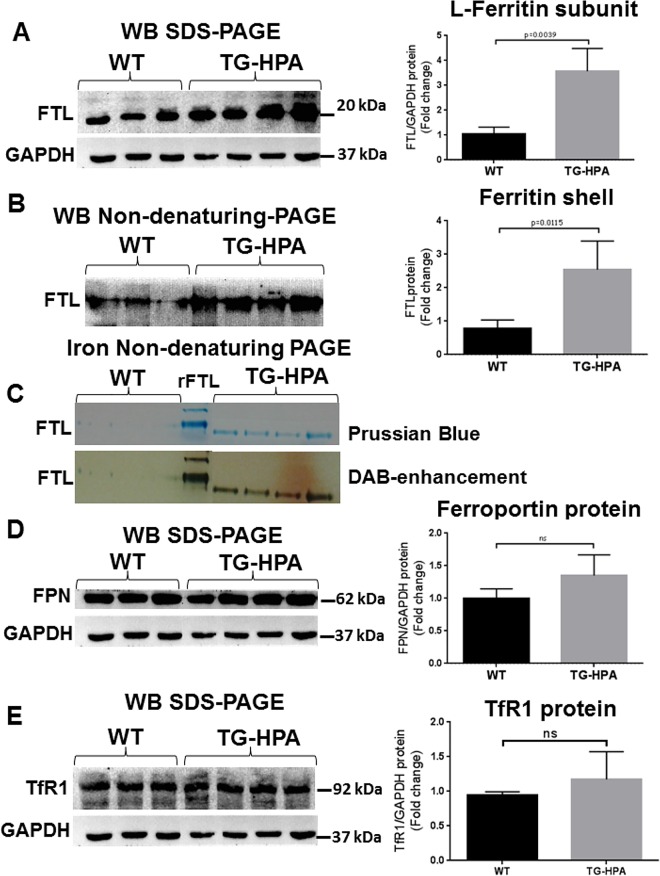
Transgenic mice overexpressing heparanase have increased ferritin-iron and ferritin protein content in the liver. (A and B) Western blot of liver extracts from WT and TG-HPA mice (A) for ferritin L-chain (FTL) subunits in SDS-PAGE with GAPDH as calibrator and (B) for assembled ferritin in non-denaturing PAGE. (C) Prussian blue stain of non-denaturing PAGE loaded with 50 ug protein, before (upper) and after enhancing with DAB and H_2_O_2_ (lower). rFTL is control purified recombinant mouse FTL. (D) Western blot of Ferroportin (FPN) and (E) of Transferrin Receptor1 (TfR1) and their respective GAPDH as calibrator. Densitometry data were obtained from 3 independent experiments.

To further characterize the liver iron overload, ferritin and iron content were evaluated in liver extracts of TG-HPA and control mice. Ferritin L-chain increased significantly in the TG-HPA mice compared to controls, when evaluated by Western blots of denaturing SDS-PAGE (Fig 6A). Western blot of total liver protein extracts separated on non-denaturing PAGE confirmed the increase of L ferritin (Fig 6B), in the TG-HPA mice. Prussian Blue staining of non-denaturing PAGE loaded with 50 μg of soluble protein of total liver extracts revealed evident ferritin-iron bands in the TG-HPA but not in the WT samples. Further enhancing the signal with H_2_O_2_/DAB confirmed the difference between the two groups of samples (Fig 6C). Unexpectedly, Ferroportin (FPN) and Transferrin receptor1 (TfR1) protein levels did not change in the TG-HPA mice, compared to control (Fig 6D and 6E). qPCR analysis of liver TfR1, Fpn and Zip14 mRNA confirmed that there are no significant changes in the expression of these iron-related genes (S4A, S4B and S4C Fig). Analyses of spleen protein extracts did not show significant differences in the content of TfR1, Ferritin L-chain and Ferroportin between TG-HPA and WT (S5A, S5B and S5C Fig). Also ferritin-iron content detected by enhanced Prussian Blue stain was analogous in TG-HPA and WT spleen (S5D Fig).

### BMP6/SMAD pathway in TG-HPA mice

**Fig 7:**
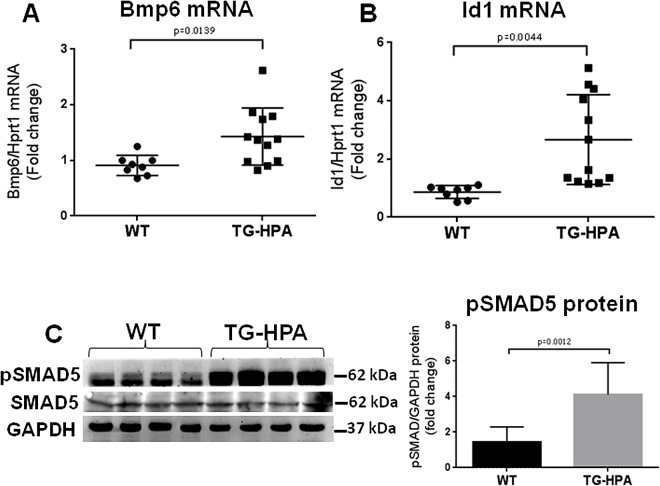
Transgenic mice overexpressing Heparanase showed increased BMP6/SMAD signaling. (A) evaluation of BMP6 mRNA in the liver of WT and TG-HPA mice (B) evaluation of Id1 mRNA, in relation to Hprt1. (C) Western blot of pSMAD5, of total SMAD5 and of GAPDH as housekeeping for normalization in densitometry quantification. The values are expressed as fold change of wild type mice.

BMP6 is an iron regulated protein and in concert with the liver iron elevation we found that BMP6 mRNA levels were significantly higher in TG-HPA mice compared to controls (Fig 7A). Unexpectedly we found that TG-HPA mice showed significantly higher Id1 mRNA levels (Fig 7B) and SMAD5 phosphorylation (4 fold) (Fig 7C) compared to control mice. These data indicated an activation of parts of the BMP6/SMAD pathway side by side with the downregulation of hepcidin in the liver of the TG-HPA mice, indicating a complex interaction of this pathway with HSPGs and iron homeostasis.

### LPS treatment in TG-HPA mice

**Fig 8:**
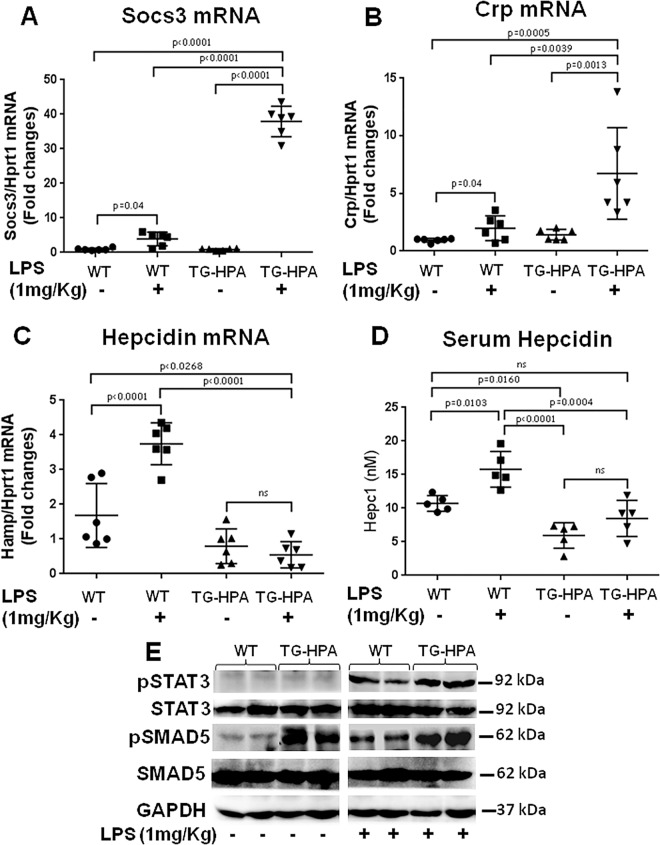
Transgenic mice overexpressing Heparanase are more sensitive to inflammatory stimuli. WT and TG-HPA mice were treated with a single dose of LPS (1 mg/Kg) IP and sacrified after 6 h. (A) Analysis of Socs3 mRNA, (B) CRP mRNA and (C) HAMP1 mRNA in the liver of WT and TG-HPA mice in relation to Hprt1, before and after the treatment. The values are expressed as fold change of wild type mice. (D) Quantification of serum Hepcidin by SELDI-TOF. (E) Western blot of pSMAD5, total SMAD5, pSTAT3, total STAT3 and of GAPDH as housekeeping.

Encouraged by the observation that the HPA clones strongly responded to inflammatory stimuli, we investigated if the same occurred in mice treated with a single dose of LPS (1 mg/kg). In the WT-mice as expected the treatment induced a significant increase of inflammation markers Socs3 and CRP (5-fold and 3-fold respectively) (Fig 8A and 8B); accompanied by a significant 3-4-fold induction of hepcidin mRNA (Fig 8C) with a significant increase of the serum protein (Fig 8D). The response of TG-HPA mice to the treatment was somewhat surprising: the induction of the inflammation markers was much stronger, 40-fold for Socs3 and 7-fold for CRP (Fig 8A and 8B), but hepcidin mRNA and serum hepcidin levels were not significantly affected by the LPS treatment (Fig 8C and 8D). Also the induction of pSTAT3 was higher in the TG-HPA than in the WT mice, whereas pSMAD5 was induced in the WT but not in the TG-HPA mice (Fig 8E). Thus, inflammation did not alter hepcidin expression in the TG mice, although it is known to be one of the major biological hepcidin inducers.

## Discussion

We have previously demonstrated that heparins have a strong anti-hepcidin activity, both *in vitro* in hepatoma cells and *in vivo* in mice, and that they act by interfering with the BMP6/SMAD pathway [9, 27]. Heparins are highly sulfated glycosaminoglycans with a structure similar to that of the endogenous HS, suggesting that HS may have an important function in the regulation of hepcidin expression and iron homeostasis. Indeed, HS can bind a number of ligands, such as cytokines, chemokines and growth factors, thereby exerting various biological functions [28]. Notably HS were recently shown to act as co-receptors for BMP2/4 in the myoblast C2C12 cell line [29], thus facilitating the formation of a multimeric BMP-receptor complex required for effective signal transduction. To verify whether a similar role of HS occurs also for BMP6-mediated signaling involved in regulation of hepatic hepcidin expression, we focused on HPA1, the main enzyme degrading HS. HPA1 is known to remodel cell surface and extracellular matrix HS, and it is over-expressed in tumor cells where it facilitates the metastatic potential, invasiveness, and neovascularization [18].

We first overexpressed heparanase in three hepatoma cells lines and found a significant suppression of hepcidin expression in all of them. Then we focused on the more familiar HepG2 cells where we confirmed that HPA expression suppressed the BMP6/SMAD signaling pathway, in both transiently transfected cells and stable clones. The induction of the 50-kDa-active HPA form ranged from 10 to 50 fold in the different transiently transfected cells, but the suppression of hepcidin mRNA remained around 50%, suggesting that it reached saturation. Similarly, despite a 10-fold difference in the exogenous HPA expression, the two stable HPA clones showed a comparable inhibition of the BMP6/SMAD pathway and of hepcidin transcription. Both clones showed an increase of ferritin protein and of ferroportin mRNA with a decrease of TfR1 protein and transcript, indicating that HPA expression caused a cellular iron excess. It is unlikely that this is direct effect of hepcidin inhibition, which would decrease rather than increase iron load. The finding may suggest a role of HS in cellular iron uptake. However the analyses of the iron transporters TfR1, ferroportin and ZIP14 did not provide indications of significant abnormalities and IRP activity could not be measured. The study of the role of HS in cellular iron homeostasis seems complex and will be considered in future research. Our data indicated that the HPA clones had also a different response to exogenous heparin. In fact a low concentration of RO-heparin had a stronger inhibitory effect on hepcidin mRNA in HPA cells than in control cells. However, the effect was rather mild, and was not observed after BMP6 stimulation. Nevertheless, it indicated that part of the inhibitory effect of exogenous heparin involved a competition with endogenous HS. We also observed that the HPA clones responded more strongly to IL6 treatment than control cells, with respect to both in Socs3 (an index of inflammatory response) and Hepcidin mRNA. HPA overexpression was shown to degrade HS which cause an increase of free cytokines and chemokines, and in turn would enhance the inflammatory response [30]. This may enhance the effect of IL6 in stimulating hepcidin, via the inflammatory pathway, which involved IL6/IL6R, the phosphorylation of STAT3 and its translocation to the nucleus to induce hepcidin mRNA transcription.

Next we analyzed the TG mice overexpressing human heparanase that have been previously described [20]. Interestingly, the expression of hepcidin mRNA in the liver was about 50% lower than that of the control mice, and also the level of hepcidin protein in serum was reduced (Fig 4A and 4B). Consistent with the low hepcidin and consequent high iron mobilization from macrophages and accumulation in parenchymal cells, the mice showed reduced spleen iron and increased liver iron. Thus, the *in vitro* and *in vivo* data fully support the notion that HS alteration caused by HPA overexpression reduces hepcidin expression, and that this affects systemic iron homeostasis. The upregulation of BMP6 mRNA can be attributed to the chronic liver iron load [31]. An unexpected finding was that the liver indices of the activation of the BMP/SMAD pathway were stimulated in TG-HPA mice, rather than repressed as in the HPA clones. For example the levels of Id1 mRNA and phospho-SMAD5 protein were increased, while in the HPA clones they were reduced. The lack of agreement between the HPA1 clones and HPA1 mice is puzzling, and suggests that HPA mice may activate factors that affect the SMADs without stimulating hepcidin expression. HS are known to retain various growth factors, cytokines and their antagonists, which can be liberated upon HPA1-mediated disruption of HS. For example, some BMP antagonists, such as Noggin, Gremlin1 and Sclerostin, are heparin binding protein [32–34] that are probably modulated by endogenous HS with a mechanism that remains to be clarified. Alternatively, the free HS produced by HPA1 overexpression may compete or interfere with the cellular HS in a non-predictable way. It should be noted that other examples in which the hepcidin expression is unrelated to BMP/SMAD activation have been reported recently. For example in Activin-B deficient mice LPS treatment stimulated hepcidin expression without activation of the SMAD pathway [35] and in a mouse model of type 2 diabetes the activation of the BMP/SMAD pathway was associated with hepcidin mRNA downregulation [36]. Also the response of the TG-HPA mice to the inflammatory stimulus was somewhat unexpected: a single injection of LPS produced a stimulation of the inflammatory indices like Socs3 and Crp mRNAs many fold higher than that of the control mice, similar to what we observed in the HPA clones stimulated by IL6. However *in vivo* this was accompanied by the lack of hepcidin induction, which instead showed a non-significant tendency to reduction. In the control mice hepcidin was upregulated by LPS, with a minor 2–3 fold induction of the two inflammatory indices.

In conclusion, we show that HPA overexpression in cells and in mice downregulates hepcidin expression, induces iron loading probably by modifying endogenous HS structure and causes an abnormal response of hepcidin to inflammation in mice, but not in the cells. This indicates a role of HS in the regulation of iron homeostasis, the clarification of which may offer novel therapeutic targets for disorders of iron metabolism and inflammation.

## Supporting Information

S1 Fig Hepatoma cells transiently transfected with heparanase showed a reduction of hepcidin mRNA. Hep3b and HuH7 cells were transfected with pcDNA3.1-HPA plasmid (HPA) or empty pcDNA3.1 as control (MOCK) and harvested 48 h after the transfection. The level of HPA mRNA and (A-C) Hepcidin mRNA (B-D) was analyzed by qPCR and normalized for Hprt1. In (A-C) the values are expressed as–dCt for HPA mRNA, in B and D as fold change over the control (MOCK) for hepcidin mRNA.
(PDF)

S2 Fig Analysis of some iron related genes in HepG2 clones overexpressing heparanase. Two stable clones of HepG2 cells transfected with pcDNA3.1-HPA (HPA3 and HPA6) were analyzed for (A) TfR1 mRNA, (B) Fpn mRNA, (C) Zip14 mRNA and (D) Bmp6 by qPCR. The values are expressed as fold change over the control (MOCK).
(PDF)

S3 Fig Treatment with heparin and BMP6 of HepG2 clones overexpressing heparanase. (A) The two HepG2 clones overexpressing HPA (HPA3 and HPA6) and control (MOCK) cells were treated with 0.12 μg/mL of RO-82 heparin in presence of BMP6 stimulation. Cells were harvested after 6 h and hepcidin mRNA evaluated in relation to Hprt1. The values are expressed as fold change of their respective controls. (B) The two HepG2 clones overexpressing HPA (HPA3 and HPA6) and control (MOCK) cells were treated with 6 and 12 ng/mL of BMP6 for 6 h. Cells were harvested and hepcidin mRNA evaluated in relation to Hprt1. The values are expressed as fold change of their respective controls.
(PDF)

S4 Fig Analysis of some iron related genes in mice overexpressing heparanase. Liver mRNA levels of (A) TfR1, (B) Fpn and (C) Zip14 were analyzed by qPCR in wild type (WT) and transgenic HPA mice (TG-HPA). The values are expressed as fold change of wild type mice and normalized to Hprt1.
(PDF)

S5 Fig Transgenic mice overexpressing heparanase showed normal levels of ferritin-iron, ferritin, Transferrin receptor and Ferroportin protein content in the spleen. Western blot of spleen extracts from WT and TG-HPA mice (A) for Transferrin Receptor1 (TfR1), (B) L-ferritin subunit (FTL) and (C) Ferroportin (FPN) in SDS-PAGE with Actin as calibrator (D) Prussian blue stain of non-denaturing PAGE loaded with 50 ug protein after enhancing with DAB and H2O2. rFTL is control purified recombinant mouse FTL. (D). Densitometry data were obtained from 3 independent experiments.
(PDF)
