# Supplementary material for: Heparanase Overexpression Reduces Hepcidin Expression, Affects Iron Homeostasis and Alters the Response to Inflammation
Source: PLoS One. 2016 Oct 6;11(10):e0164183. doi: 10.1371/journal.pone.0164183 (PMC5053418; doi:10.1371/journal.pone.0164183)
Supplement: S1 Fig — Hep3b and HuH7 cells were transfected with pcDNA3.1-HPA plasmid (HPA) or empty pcDNA3.1 as control (MOCK) and harvested 48 h after the transfection. The level of HPA mRNA and (A-C) Hepcidin mRNA (B-D) was analyzed by qPCR and normalized for Hprt1. In (A-C) the values are expressed as–dCt for HPA mRNA, in B and D as fold change over the control (MOCK) for hepcidin mRNA. (PDF) [file pone.0164183.s001.pdf]

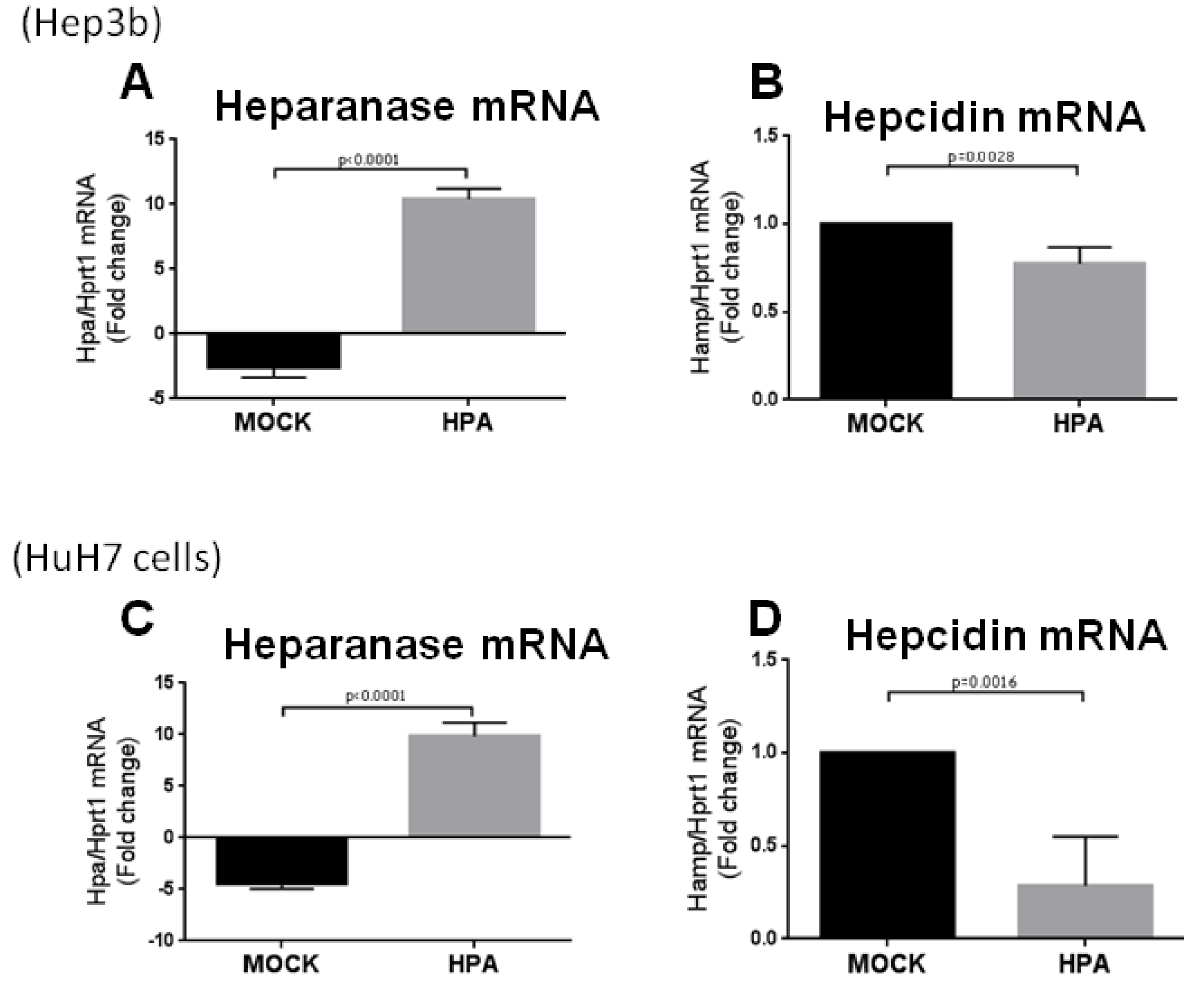

S1 Fig. Hepatoma cells transiently transfected with heparanase showed a reduction of hepcidin mRNA. Hep3b and HuH7 cells were transfected with pcDNA3.1-HPA plasmid (HPA) or empty pcDNA3.1 as control (MOCK) and harvested 48 h after the transfection. The level of HPA mRNA and (A-C) Hepcidin mRNA (B-D) was analyzed by qPCR and normalized for Hprt1. In (A-C) the values are expressed as –dCt for HPA mRNA, in B and D as fold change over the control (MOCK) for hepcidin mRNA.
