# Supplementary material for: Heparanase Overexpression Reduces Hepcidin Expression, Affects Iron Homeostasis and Alters the Response to Inflammation
Source: PLoS One. 2016 Oct 6;11(10):e0164183. doi: 10.1371/journal.pone.0164183 (PMC5053418; doi:10.1371/journal.pone.0164183)
Supplement: S2 Fig — Two stable clones of HepG2 cells transfected with pcDNA3.1-HPA (HPA3 and HPA6) were analyzed for (A) TfR1 mRNA, (B) Fpn mRNA, (C) Zip14 mRNA and (D) Bmp6 by qPCR. The values are expressed as fold change over the control (MOCK). (PDF) [file pone.0164183.s002.pdf]

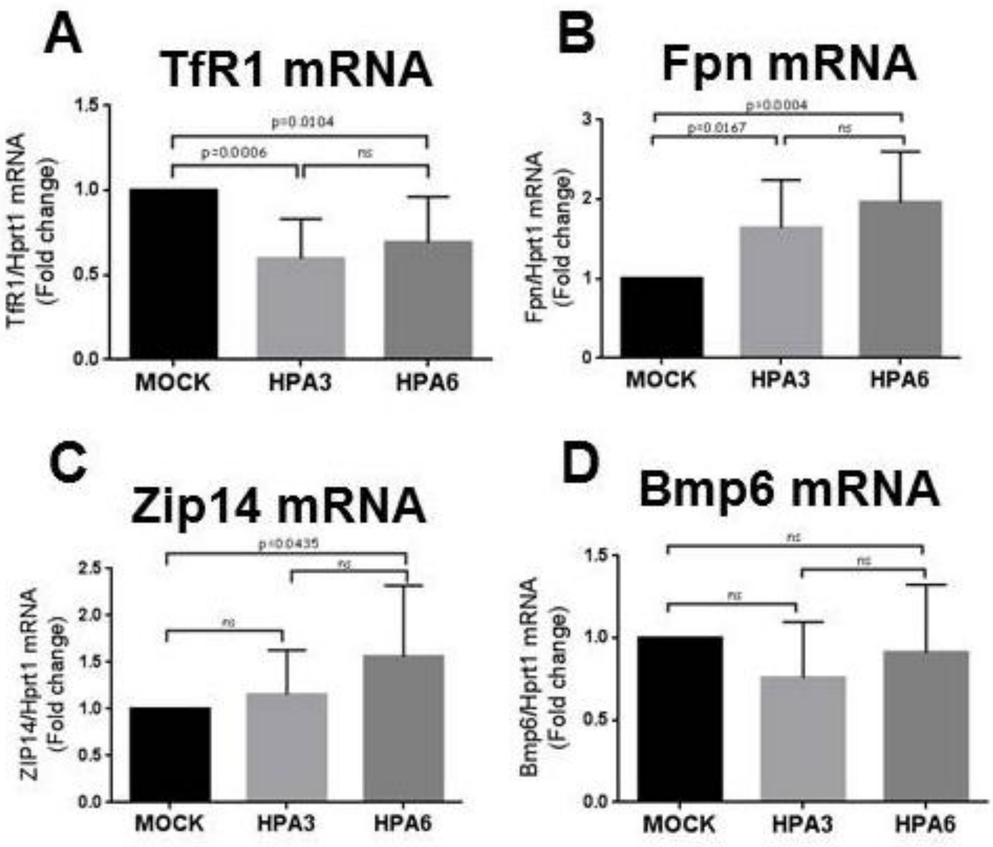

S2 Fig.Analysis of some iron related genes in HepG2 clones overexpressing heparanase.
Two stable clones of HepG2 cells transfected with pcDNA3.1-HPA (HPA3 and HPA6) were analyzed for (A) TfR1 mRNA, (B) Fpn mRNA, (C) Zip14 mRNA and (D) Bmp6 by qPCR. The values are expressed as fold change over the control (MOCK).
