# Supplementary material for: Heparanase Overexpression Reduces Hepcidin Expression, Affects Iron Homeostasis and Alters the Response to Inflammation
Source: PLoS One. 2016 Oct 6;11(10):e0164183. doi: 10.1371/journal.pone.0164183 (PMC5053418; doi:10.1371/journal.pone.0164183)
Supplement: S3 Fig — (A) The two HepG2 clones overexpressing HPA (HPA3 and HPA6) and control (MOCK) cells were treated with 0.12 μg/mL of RO-82 heparin in presence of BMP6 stimulation. Cells were harvested after 6 h and hepcidin mRNA evaluated in relation to Hprt1. The values are expressed as fold change of their respective controls. (B) The two HepG2 clones overexpressing HPA (HPA3 and HPA6) and control (MOCK) cells were treated with 6 and 12 ng/mL of BMP6 for 6 h. Cells were harvested and hepcidin mRNA evaluated in relation to Hprt1. The values are expressed as fold change of their respective controls. (PDF) [file pone.0164183.s003.pdf]

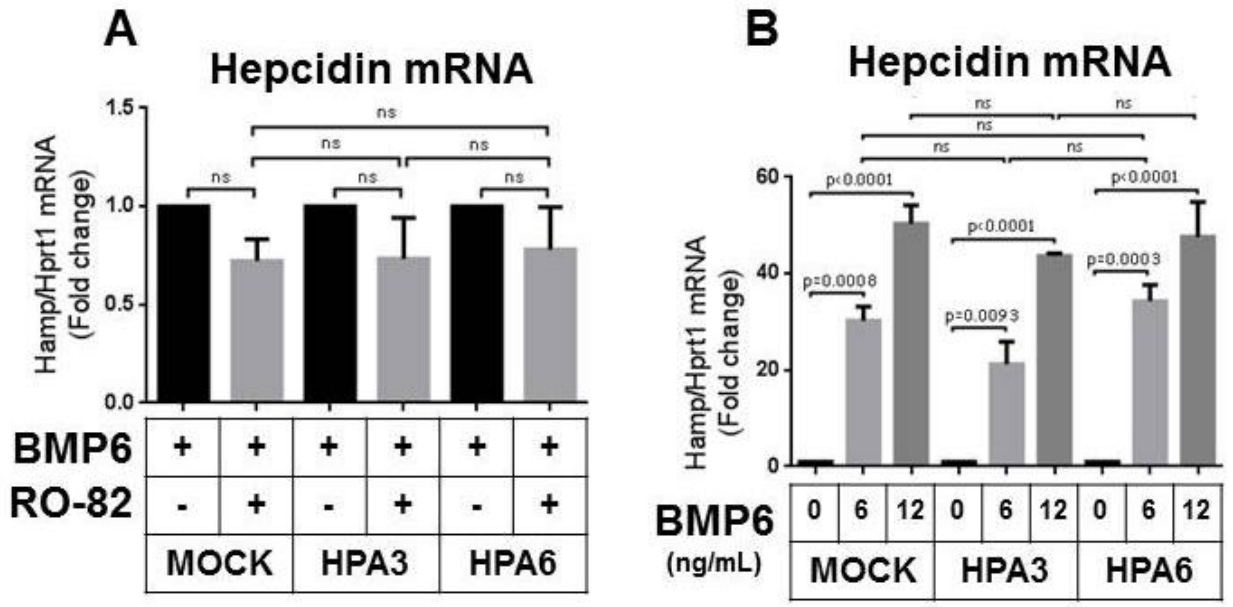

S3 Fig. Treatment with heparin and BMP6 of HepG2 clones overexpressing heparanase. (A) The two HepG2 clones overexpressing HPA (HPA3 and HPA6) and control (MOCK) cells were treated with 0.12 µg/mL of RO-82 heparin in presence of BMP6 stimulation. Cells were harvested after 6 h and hepcidin mRNA evaluated in relation to Hprt1. The values are expressed as fold change of their respective controls. (B) The two HepG2 clones overexpressing HPA (HPA3 and HPA6) and control (MOCK) cells were treated with 6 and 12 ng/mL of BMP6 for 6 h. Cells were harvested and hepcidin mRNA evaluated in relation to Hprt1. The values are expressed as fold change of their respective controls.
