# Supplementary material for: Heparanase Overexpression Reduces Hepcidin Expression, Affects Iron Homeostasis and Alters the Response to Inflammation
Source: PLoS One. 2016 Oct 6;11(10):e0164183. doi: 10.1371/journal.pone.0164183 (PMC5053418; doi:10.1371/journal.pone.0164183)
Supplement: S4 Fig — Liver mRNA levels of (A) TfR1, (B) Fpn and (C) Zip14 were analyzed by qPCR in wild type (WT) and transgenic HPA mice (TG-HPA). The values are expressed as fold change of wild type mice and normalized to Hprt1. (PDF) [file pone.0164183.s004.pdf]

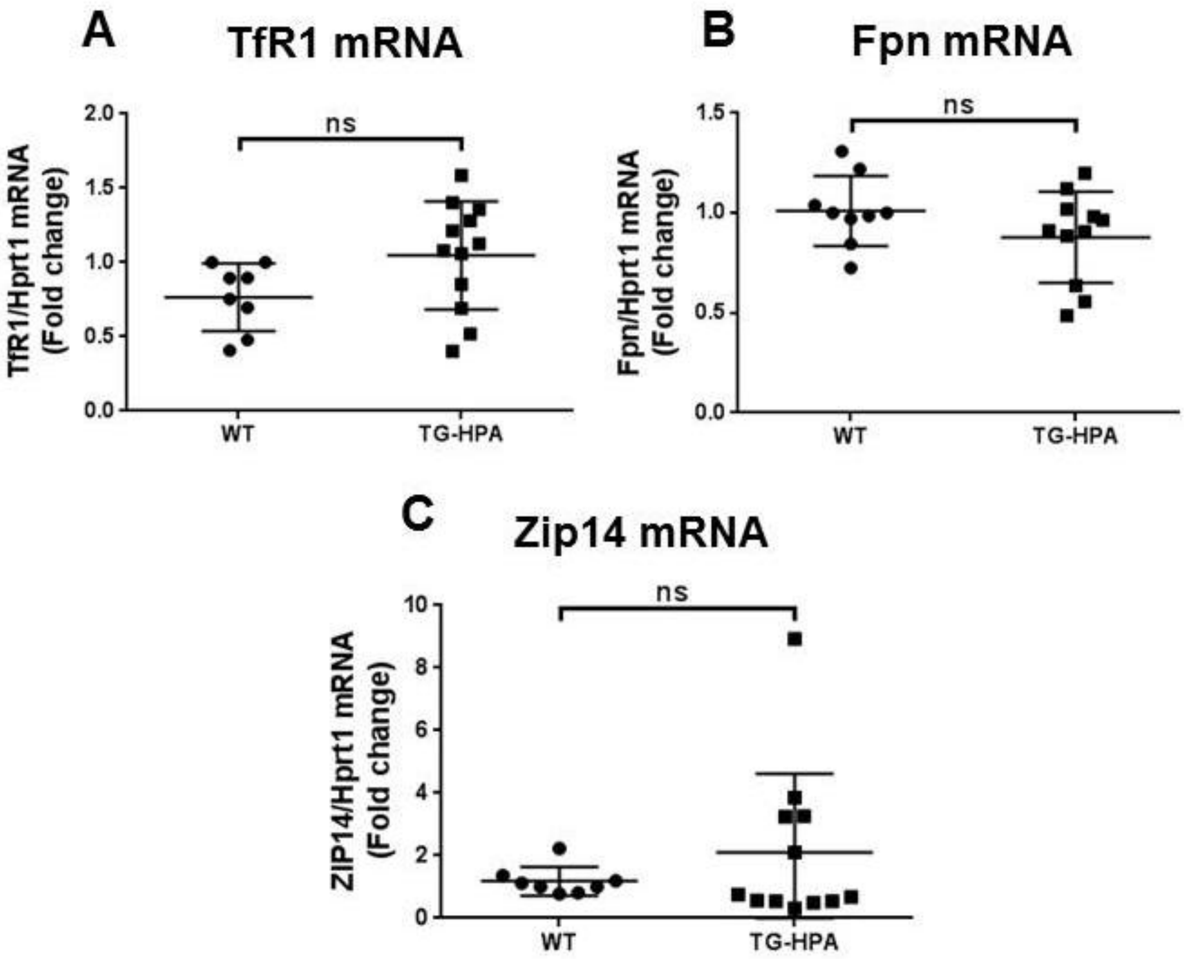

S4 Fig. Analysis of some iron related genes in mice overexpressing heparanase.
Liver mRNA levels of (A) TfR1, (B) Fpn and (C) Zip14 were analyzed by qPCR in wild type (WT) and transgenic HPA mice (TG-HPA). The values are expressed as fold change of wild type mice and normalized to Hprt1.
