# Supplementary material for: Heparanase Overexpression Reduces Hepcidin Expression, Affects Iron Homeostasis and Alters the Response to Inflammation
Source: PLoS One. 2016 Oct 6;11(10):e0164183. doi: 10.1371/journal.pone.0164183 (PMC5053418; doi:10.1371/journal.pone.0164183)
Supplement: S5 Fig — Western blot of spleen extracts from WT and TG-HPA mice (A) for Transferrin Receptor1 (TfR1), (B) L-ferritin subunit (FTL) and (C) Ferroportin (FPN) in SDS-PAGE with Actin as calibrator (D) Prussian blue stain of non-denaturing PAGE loaded with 50 ug protein after enhancing with DAB and H2O2. rFTL is control purified recombinant mouse FTL. (D). Densitometry data were obtained from 3 independent experiments. (PDF) [file pone.0164183.s005.pdf]

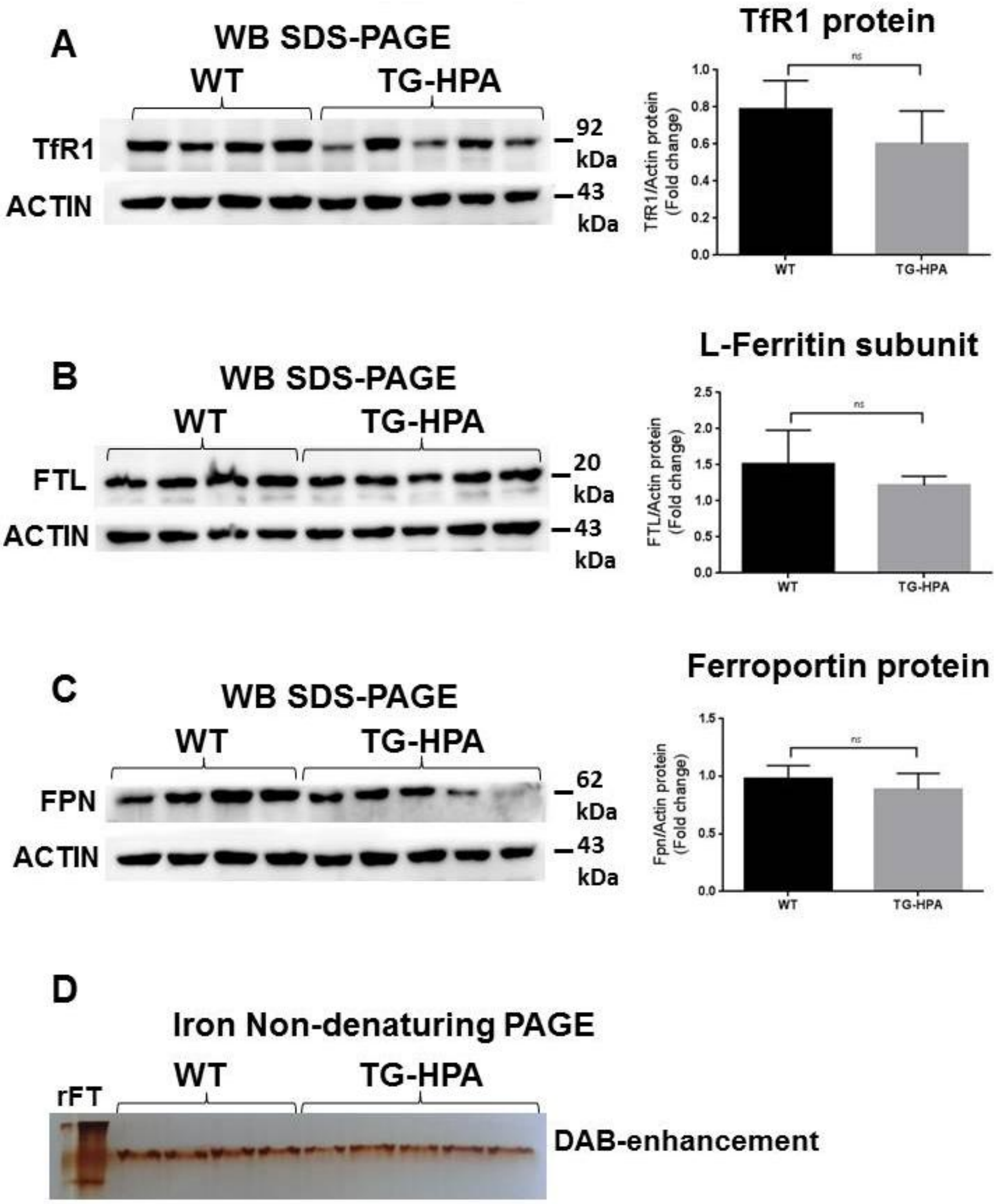

S5 Fig. Transgenic mice overexpressing heparanase showed normal levels of ferritin-iron, ferritin, Transferrin receptor and Ferroportin protein content in the spleen. Western blot of spleen extracts from WT and TG-HPA mice (A) for Transferrin Receptor1 (TfR1), (B) L-ferritin subunit (FTL) and (C) Ferroportin (FPN) in SDS-PAGE with Actin as calibrator (D) Prussian blue stain of non-denaturing PAGE loaded with 50 ug protein after enhancing with DAB and H2O2. rFTL is control purified recombinant mouse FTL. (D). Densitometry data were obtained from 3 independent experiments.
